# Advances in Porous Materials and Their Applications

**DOI:** 10.1021/acscentsci.6c01286

**Published:** 2026-08-26

**Authors:** Seth M. Cohen

**Affiliations:** Department of Chemistry, University of California, Irvine, Irvine, California 92697, United States

The development of new porous materials has seen a revolution and rapid expansion over the last ∼ 25 years. One could argue that this year might be considered the ‘Year of the Porous Material’ thanks to the awarding of the 2025 Nobel Prize in Chemistry to Robson, Kitagawa, and Yaghi for their collective discovery of metal–organic frameworks (MOFs). Comprised of inorganic metal ion clusters and coordinating organic ligands, MOFs have led this recent renaissance and transformation in porous materials. *ACS Central Science* recently published an Outlook article by **Morris** and **Shustova**, celebrating the Nobel prize winning discovery, including insights into the present state and future of the field (10.1021/acscentsci.6c00758). However, MOFs are certainly not the only new porous materials that have emerged in the last several decades. Alongside *metal–organic frameworks (MOFs)*, other porous materials, including *covalent organic frameworks (COFs)*, *hydrogen-bonded frameworks (HOFs)*, *porous organic polymers (POPs)*, *polymers of intrinsic microporosity (PIMs)*, and other creative constructs that manipulate molecules into creating unprecedented levels of open space within their confines have emerged. Although many researchers will rightly point to the rich chemistry of zeolites and porous carbons that predate (in both discovery and application) the more contemporary materials mentioned above, it is hard to deny the vast structural diversity, chemical intricacy, functional richness, and expansive surface areas these newer materials have to offer. The ability to achieve not only porosity, but also pore tuning (the subject of a prior Editorial: 10.1021/acscentsci.3c00916), is one of the truly exceptional features of these ‘next-generation’ porous materials.

This Collection of recently published studies in *ACS Central Science* highlights the importance of this materials revolution, coinciding with the 2025 Nobel Prize recognition. These articles represent continuing fundamental advancements in the field, the increasing role of AI/ML, as well as demonstrations of the potential applications of these materials in many fields of science. In the opinion of this author, the real-world, emerging applications of MOFs were an important factor in MOFs being elevated to our discipline’s highest honor (although it should be noted that the author was not on the Nobel selection committee and any insight is purely speculative). It is hoped that this collection will help guide interested readers to some of the latest, most cutting-edge advancements in the field being published in *ACS Central Science*.
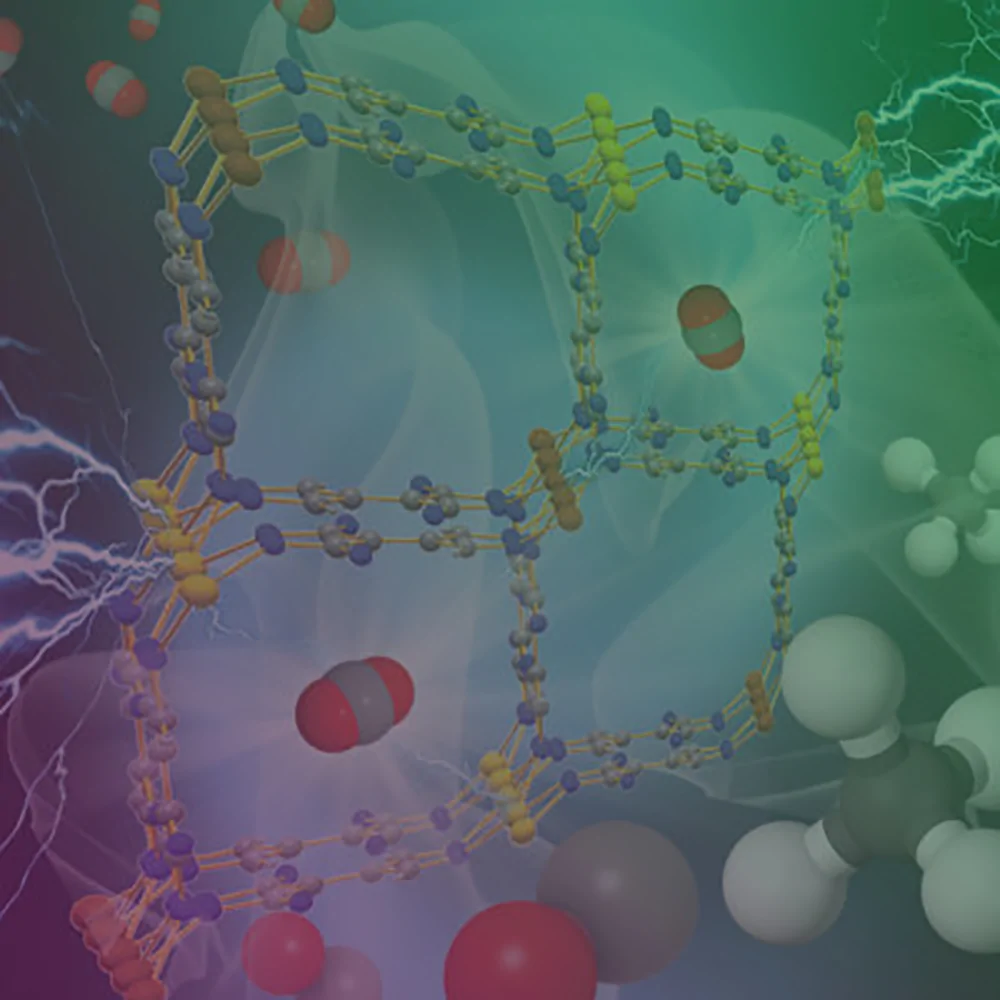


## Water Capture

One application of next-generation porous solids that has grown dramatically in the past decade is the area of atmospheric water capture. The high surface areas and tunable water-binding isotherms that can be achieved by the design and functionalization of MOFs and other porous solids has brought about renewed excitement around the possibility of harvesting water from ambient air. The ability to capture air, even from very arid climates, has important applications ranging from humanitarian relief to military operations. Indeed, the author initiated a program at the Defense Advanced Research Projects Agency (DARPA) entitled Atmospheric Water Extraction (AWE) to address the materials chemistry and engineering challenges associated with developing water capture devices, many of which have been motivated by the power of next-generation porous materials to adsorb water. Indeed, one of the articles in this collection was supported by the AWE program, where **Siepmann**, **Gagliardi**, and **Yaghi** utilize linker extension to enhance the water uptake properties of MOFs. Linker extension has been long used in MOFs as a general strategy for achieving ever higher surface areas, but in the design of materials for water capture there are additional considerations. Specifically, there remains the challenge of increasing water uptake capacity, while not disrupting the ability of the materials to take up water at low relative humidities. The authors address this problem by using a computationally driven ligand design that identifies the right balance of hydrophobicity and hydrophilicity to maximize both desired attributes (10.1021/acscentsci.3c00018). In another study that combines experimental and computational investigations, **Sauer**, **Gagliardi**, and **Yaghi** describe an approach that allows for prediction and tuning of water uptake properties in COFs (and MOFs). The authors devise a ‘hydrophilicity index’ that can be related to the ‘step’ in the water adsorption isotherms in these materials (10.1021/acscentsci.4c01878). Using a different porous material, **Zhang** and **Liu** tune the water sorption behavior of HOFs (10.1021/acscentsci.5c01233). In their study, the authors employ a popular strategy from MOF chemistry, the concept of ‘multivariate’ (MTV) materials, where the mixing of distinct, but structurally compatible building blocks is used to create a porous material with tunable functionality, and hence tunable characteristics. In this case an ‘unfunctionalized’ [1,1′:4′,1″-terphenyl]-3,3″,5,5″-tetracarboxylic acid (TPTCA) synthon is combined with an otherwise identical, but amino-functionalized synthon to prepare mixed-synthon HOFs. These materials, which could be referred to as ‘MTV-HOFs’ could be prepared with tunable levels of amino-groups that allowed for tuning of water sorption characteristics, including the step in the water adsorption isotherm. It is a testament to these porous materials that a framework solid connected via hydrogen bonds can be sufficiently robust to act as a medium for water capture.

## Catalysis

The use of next-generation porous materials as catalysis has been another active area of research (10.1021/acscentsci.2c01083). Early discussions on MOFs speculated on the ability to tune the pores of these materials with programmable sizes, shapes, chirality, and functionality. The ability to program pore hydrophobicity, hydrogen-bonding, acidic or basic groups, and active metal centers frequently raised the question of whether MOFs and other porous solids could exhibit biomimetic catalysis. Indeed, many porous solids have exhibited exciting catalytic activity, with biomimetic like behavior and beyond. **Tang** and **Ma** describe their enzyme-inspired catalysis within the pores of a MOF that harnesses chiral Frustrated Lewis Pairs (FLPs), a distinctively abiologic functional group for achieving catalysis (10.1021/acscentsci.3c00637). Motivated by the activity of reductase enzymes, they prepare a MOF that can hydrogenate α,β-unsaturated imines to produce chiral β-unsaturated amines in good yields with high enantioselectivity and chemoselectivity. The ability to tune the composition and reactivity of the secondary-building units (SBUs), the inorganic building blocks of MOFs, is another opportunity for introducing catalytic activity. In that vein, **Liu** and **Huang** compare Zr­(IV) and Hf­(IV) SBUs in otherwise isostructural MOFs using experimental, spectroscopic, and computational methods (10.1021/acscentsci.2c01140). Their studies show that the change from one metal to the other can dial up or down Bro̷nsted vs Lewis acidity, which defines the catalytic characteristics of the material. Beyond framework-derived catalysis, next-generation porous solids have also been used to encapsulate catalytic species, including enzymes. For example, **Yuan**, **Zheng**, and **Cheng** report on a micelle-controlled synthesis of a MOF that can encapsulate a lipase, which then can perform efficient, enantioselective catalysis on a drug precursor substrate (10.1021/acscentsci.3c01432).

Beyond MOFs, **Wang** and **Ma** describe the photocatalytic characteristics of COFs (10.1021/acscentsci.5c01645). A number of COFs share common features, such as consisting of 2-dimensional, hexagonal topologies comprised of rigid, aromatic building blocks. As such, many possess broad absorption features, which can be harnessed for photochemical applications, including photocatalysis. In this study, the authors demonstrate that electronic conjugation can influence the distribution of photoelectrons in the COF, while induction effects can direct these reducing equivalents to specific sites. This allows for tuning of the electron–hole separation and transport to enhance photocatalytic activity. The authors demonstrate the application of these COF photocatalysts in the reductive separation of uranium ions from spiked ground- and seawater. **Zhang** also explores COFs as photocatalysts for uranium extraction (10.1021/acscentsci.3c01195). In their example, the authors use an elegant synthetic strategy to create a layered COF structure comprised of imine- and vinyl-linked 2-dimensional COF layers. The resulting ‘layer-blocked’ structure enhances the stability of the photocatalytic system.

In a related (but not explicitly catalytic) study, **Zhao** and **Zhu** examine electrochemical extraction of uranium using porous aromatic framework (PAF, which might also be classified as POPs) electrodes (10.1021/acscentsci.3c01291). POPs/PAFs constitute a less crystalline, less well ordered porous solid when compared to MOFs and COFs, but nonetheless represent another group of important, next-generation porous solids. Indeed, the investigation of next-generation porous solids as conductive materials and in electrochemical (and photoelectrochemical) applications is growing at a rapid pace, as illustrated by the other reports in this Collection led by **Unwin** and **Dinča** (10.1021/acscentsci.2c00509), **Chao** and **Zhao** (10.1021/acscentsci.5c00365), and **Hua** and **Zhao** (10.1021/acscentsci.4c00416).

## Infusion of Computation and AI

As with so many disciplines today, both within and without science, machine learning (ML) and artificial intelligence (AI) are beginning to make their mark on the future of porous solids. This Collection features several articles that use these tools for the discovery, prediction, and advancement of porous solids. In one study, **Kim** applies a model for quantum computing to predict material characteristics of MTV-MOFs (10.1021/acscentsci.5c00918). As mentioned above with respect to HOFs, MTV-MOFs are ‘solid solutions’ that contain mixtures of building blocks (typically mixtures of differentially functionalized organic building blocks) within a single crystalline lattice. In this study, the authors optimize MTV-MOF design using advanced computational methods, while acknowledging the synthetic challenges with achieving such systems. This may be an area where computation is still ahead of what synthesis can achieve, presenting an exciting challenge for experimentalists in the field to tackle. Also focusing on the power of quantum, **Sriram**, **Medford**, and **Sholl** focus on one of the longstanding potential applications of porous solids, direct air capture (DAC) of carbon dioxide (CO_2_). These authors employ millions of quantum chemistry calculations to support ML models for DAC by MOFs (10.1021/acscentsci.3c01629). In their study, they create a data set with adsorption energies for mixtures of CO_2_ and H_2_O. Similarly, **Rajendran** and **Woo** also focus on computational predictions for CO_2_ capture, but in this case for flue gas capture (10.1021/acscentsci.5c00777). Their simulations identified MOFs that could retain CO_2_ even under high humidity conditions, where water is a problematic competitive binder for CO_2_ capture with these materials.

For your typical, noncomputational chemist, popular knowledge around computing and AI are associated with ChatGPT, Claude, and similar tools that are now widely available. The author suspects many of us in the field use these new tools for image generation, accelerating literature searches, and other, relatively simple tasks. However, it is becoming increasingly clear that more rigorous implementation of these tools can lead to very meaningful advancements, efficiencies, and contributions to research. In a modern example of the AI era, **Yaghi** incorporates ChatGPT into their experimental methods to identify conditions that allow for the rapid, microwave-based synthesis of MOFs and COFs (10.1021/acscentsci.3c01087). Specifically, the authors use large language model-based assistants in their chemistry workflow (which they designate as a ‘ChatGPT Research Group’) to optimize microwave synthesis conditions, for MOFs and COFs of interest for water capture (see above). It is this kind of implementation of these emerging tools that could greatly accelerate materials research. The author would argue that those who fail to familiarize themselves with these tools may find themselves falling behind a new, unexpected pace of research enabled by AI. However, like any tool, proper use of AI is critical to obtaining useful and accurate (i.e., ‘hallucination’ free) outcomes. With that in mind, **Wang** and **Yang**, discuss the importance of prompt engineering in the use of AI for porous solids research (10.1021/acscentsci.4c01935). Their Outlook highlights the present knowledge gap for many chemists in how to best use AI tools. The authors provide guidelines for prompt engineering with topical examples on MOFs. The study highlights how many of us in the chemical sciences must go ‘back to school’ so that we can use these tools to maximal effect in our research and process of discovery.

In closing, we hope our readership enjoys this collection of articles that celebrates the ‘Year of the Porous Material’, including not just the Nobel-recognized MOFs, but also COFs, HOFs, PAFs, PIMs, and the many other acronym-friendly materials that are on the rise. The importance of new materials in addressing challenges in energy, health, catalysis, and other applications elevates the potential impact of these next-generation porous solids. We encourage the community to submit their best, most innovative studies on porous solids to *ACS Central Science* to further expand the exciting repertoire of such reports in the journal.

